# Construction and Validation of a Scale to Measure Loneliness and Isolation During Social Distancing and Its Effect on Mental Health

**DOI:** 10.3389/fpsyt.2022.798596

**Published:** 2022-04-05

**Authors:** Marthe Gründahl, Martin Weiß, Lisa Maier, Johannes Hewig, Jürgen Deckert, Grit Hein

**Affiliations:** ^1^Translational Social Neuroscience Unit, Department of Psychiatry, Psychosomatics and Psychotherapy, Center of Mental Health, University of Würzburg, Würzburg, Germany; ^2^Institute of Psychology, Department of Psychology I: Differential Psychology, Personality Psychology and Psychological Diagnostics, University of Würzburg, Würzburg, Germany

**Keywords:** loneliness, social isolation, social distancing, depression, anxiety

## Abstract

A variety of factors contribute to the degree to which a person feels lonely and socially isolated. These factors may be particularly relevant in contexts requiring social distancing, e.g., during the COVID-19 pandemic or in states of immunodeficiency. We present the Loneliness and Isolation during Social Distancing (LISD) Scale. Extending existing measures, the LISD scale measures both state and trait aspects of loneliness and isolation, including indicators of social connectedness and support. In addition, it reliably predicts individual differences in anxiety and depression. Data were collected online from two independent samples in a social distancing context (the COVID-19 pandemic). Factorial validation was based on exploratory factor analysis (EFA; Sample 1, *N* = 244) and confirmatory factor analysis (CFA; Sample 2, *N* = 304). Multiple regression analyses were used to assess how the LISD scale predicts state anxiety and depression. The LISD scale showed satisfactory fit in both samples. Its two state factors indicate being *lonely and isolated* as well as *connected and supported*, while its three trait factors reflect general *loneliness and isolation, sociability and sense of belonging*, and *social closeness and support*. Our results imply strong predictive power of the LISD scale for state anxiety and depression, explaining 33 and 51% of variance, respectively. Anxiety and depression scores were particularly predicted by low dispositional *sociability and sense of belonging* and by currently being more *lonely and isolated*. In turn, being *lonely and isolated* was related to being less *connected and supported* (state) as well as having lower *social closeness and support* in general (trait). We provide a novel scale which distinguishes between acute and general dimensions of loneliness and social isolation while also predicting mental health. The LISD scale could be a valuable and economic addition to the assessment of mental health factors impacted by social distancing.

## Introduction

Increases in loneliness (i.e., a subjective lack of social connection) and social isolation (i.e., an objective lack of social interactions) have repeatedly been listed among important risk factors for mental and somatic illness [e.g., (1–3)]. The prevalence of loneliness in the population is high, with 15–30% experiencing chronic, long-term (trait) loneliness and 60–80% experiencing occasional, short-term (state) loneliness (1). Therefore, it is crucial to detect high levels of loneliness early and to intervene as soon as possible, as loneliness is associated with mental health issues such as stress, depression, anxiety, self-harm, and even suicide (4–6).

At present, several well-established measures of loneliness and isolation exist, such as the De Jong Gierveld Loneliness Scale (7) and the UCLA Loneliness Scale (8, 9). These scales measure loneliness and isolation based on the dispositional need for social contact, but do not differentiate this trait from currently felt (i.e., state) loneliness. This state-trait distinction is often overlooked in previous literature, yet important (10, 11). Although both have been related to diminishments in mental health (10, 12, 13), state and trait loneliness appear to be related to different aspects of mental health. That is, while trait loneliness is a stable predictor of mental health outcomes [i.e., anxiety and depression; (1, 10, 14)], state loneliness can predict the individual reaction to a specific incidence of social isolation, e.g., social distancing (15, 16). In general, predictions of mental health are worse for a person scoring high on trait loneliness than for a person scoring low on trait loneliness (1, 10, 14), but this does not necessarily mean that this person suffers more from acute (state) loneliness. Instead, it is possible that long-term (trait) lonely people are better equipped to cope with acute situations of loneliness and social isolation because they are used to a lack of social contact. In contrast, for a person with low trait loneliness, acute (state) loneliness may incite stronger mental health problems because this person is used to the stabilizing effect of social contact and support. A mere assessment of trait loneliness [based on existing questionnaires (7–9)] only predicts mental health in general, regardless of reactions to acute lack of social contact in a given situation. A mere assessment of state loneliness only predicts situational mental health outcomes in reaction to an acute lack of social contact (e.g., due to social distancing), regardless of the individual's dispositional need for and access to social contact. Thus, our studies focused on developing an instrument that assesses loneliness and isolation on both the state and the trait level, and tested its predictive power for important dimensions of mental health, i.e., anxiety and depression.

The necessity to assess loneliness and isolation on the state and trait level is particularly obvious in times of social distancing, i.e., during the reduction of physical proximity and direct social contacts. Social distancing is prominent in the current COVID-19 pandemic (17–19), but is also used in many other clinical contexts to prevent virus or disease transmission, e.g., in response to seasonal influenza outbreaks (20, 21) or as a pre-emptive intervention for severely ill individuals (22, 23). For instance, patients suffering from cancer (24) or severe immunodeficiency (25) may need to socially distance from others to protect their weakened immune system from additional strain. Social distancing could even refer to solitary confinement, which in essence corresponds to isolating a person from contact with others (26). Social distancing in all its forms can enhance loneliness and related declines in mental health (15, 16, 19, 27). It prevents individuals from fulfilling their need for social contact and connectedness, thus increasing feelings of loneliness and social isolation (15, 28). To sensitively predict the effect of social distancing-induced loneliness, social isolation and mental health, both state and trait dimensions need to be assessed and put in relation to each other.

Previous literature has presented loneliness and social isolation as either convergent or distinct constructs (29, 30). Social isolation can be divided into two aspects that focus on objective and subjective aspects of isolation, respectively: social disconnectedness and perceived isolation. Objective aspects (social disconnectedness) focus on physical separation from others, i.e., the observable absence of social contacts. Subjective aspects (perceived isolation) capture how a person perceives the (un)availability of social support, companionship, and emotional closeness to others (31, 32). Similar to perceived isolation and sometimes used synonymously (33), the concept of loneliness refers to the subjective dimensions of social isolation, to feeling disconnected and lacking meaningful companionship and integration. One's social relationship network is perceived as inadequate, and there is a discrepancy between the desire for social connection and the perception of one's actual relationships (34). Based on these theoretical similarities, this study presumes a convergent and fluent conceptualization of perceived isolation and loneliness. Thus, we focus on these subjective perceptions of loneliness and social isolation in the context of social distancing, i.e., during an objective reduction in social contacts. Notably, physical separation from others does not necessarily lead to loneliness. Instead, there are important inter-individual differences to this relation (29). For instance, those who have small social networks or rarely participate in social activities still might not feel lonely if the contacts they have match their needs. At the same time, people may be socially active and part of several social groups, but nevertheless feel lonely, left out or isolated, if their relationships lack emotional closeness and support (1, 29, 31, 34).

Social isolation, loneliness and their effect on mental health relate to a number of factors. As presented above, the perception of social support, i.e., having people to rely on who may provide care, value and love (35), is an important aspect in the definition and measurement of loneliness and social isolation (8, 31, 34). In social distancing contexts, lower levels of social support are associated with increases in loneliness (28, 35–37). In general, perceived social support, stable social relationships and face-to-face interactions have been found to reduce loneliness and isolation (31, 37) and enhance mental health (38–41). Another important factor are individual differences in extraversion, a personality trait representing sociability and the enjoyment and appreciation of engaging in social contacts (42–44). Higher extraversion relates to lower levels of loneliness (44) and positive mental health, including psychological and social wellbeing (45). In contrast, anxiety in social interactions and related avoidance of social situations are associated with decreases in mental health (46, 47) and increases in loneliness (48). However, during social distancing, those with a predisposition to seek social engagement (i.e., extraverts) might suffer more from limited access to direct social contacts, resulting in higher state loneliness and isolation (49). At the same time, a dispositional tendency to avoid social contacts (i.e., social anxiety) could prevent increases in state loneliness and isolation, as social contact restrictions agree with dispositional tendencies to avoid contacts. Thus, the relation of extraversion and social anxiety with loneliness, isolation and mental health during social distancing is unclear.

The effect of social distancing on loneliness and isolation could depend on its extent. In times of social distancing, continued access to close contacts should protect against loneliness and impaired mental health (36, 50). As social distancing particularly targets distancing from high-risk persons (51), individuals who were regularly in contact to high-risk persons before the social distancing context might particularly miss these contacts, leading to an increase in loneliness. Virtual communication and virtual interactions could be an important substitute for face-to-face contacts during social distancing. Virtual interactions were shown to reduce both loneliness and depression (52) and provide an alternative medium to maintain social support and a sense of belonging in social distancing contexts and in general (53, 54). Finally, gender and age should be considered when investigating loneliness, social isolation and mental health. Younger age leads to a higher prevalence in anxiety (55, 56) and depression, at least in high-income countries (57, 58). Regarding loneliness and age, findings are ambiguous, implying either a decrease (59), a U-shaped relation (lowest level in middle-aged individuals) (60), or an increase (61) of loneliness with age. Regarding gender, previous findings imply a lower prevalence of loneliness (59), but a higher prevalence for depression and anxiety (62–64) in women compared to men.

Previous research indicates that loneliness is a risk factor for later loneliness, social isolation, and impaired social functioning and connectedness (1, 10, 14). It is therefore likely that the degree of acute (state) loneliness and isolation caused by social distancing is affected by pre-existing (trait) loneliness and isolation and (lack of) access to social support and integration. Next to these dispositional factors, current access to social support could reduce acute loneliness and isolation. Given its strong predictive relation with mental health measures, a reliable state-trait measure of loneliness and isolation in the context of social distancing is vital to detect and predict negative psychological effects of social distancing measures. An economic yet comprehensive assessment with one single instrument would facilitate timely intervention to protect and enhance wellbeing and prevent long-term health consequences (65).

Here, we introduce the Loneliness and Isolation during Social Distancing (LISD) Scale which differentiates between state and trait variables. Its two subscales assess (1) general feelings of loneliness and social isolation along with the dispositional need and availability of social contacts and support (i.e., on the trait level) and (2) acute feelings of loneliness and isolation along with the current situational context, including social contacts and support (i.e., on the state level). The LISD scale was validated during a social distancing context (i.e., the COVID-19 pandemic) and linked to important dimensions of mental health (i.e., anxiety and depression).

We hypothesized that high scores in LISD traits assessing loneliness and isolation are positively related to LISD state scores representing loneliness and isolation during social distancing. That is, the stronger a person's general feelings of loneliness and isolation and related dispositional need for social contact, the stronger (i.e., the more negative) this person's acute loneliness and isolation. At the same time, LISD traits representing social support and connectedness should relate negatively to these LISD state scores. The stronger the perceived social support and connectedness, the lower the state loneliness and isolation. Moreover, greater habitual and acute use of virtual interactions, and more stable access to social contacts (trait and state) should also reduce state loneliness and isolation.

The relationship between state and trait loneliness and isolation may be influenced by individual differences in extraversion and social anxiety. Inspired by previous literature (49), we predicted that extraverts may be more negatively affected by social distancing due to their preference for social participation, resulting in higher state loneliness and isolation. In contrast, as social anxiety is related to the tendency to avoid social contacts (46), socially anxious individuals may show lower levels of loneliness and isolation.

Finally, we hypothesized that both state and trait indicators of loneliness and isolation predict mental health outcomes during acute situations of social distancing. Thus, higher scores on loneliness and isolation were expected to relate to higher self-rated state anxiety and depression. Indicators of social withdrawal and avoidance were also expected to relate to higher anxiety and depression scores. In contrast, higher levels of perceived social support, perceived social connectedness and extraversion as well as continued access to social contacts should relate to lower anxiety and depression.

## Methods

### Construction of the LISD Scale

Scale construction followed a deductive approach. Items were selected based on literature research, already established instruments, and our own theoretical considerations [see e.g., (66)]. Two researchers (first and last author) pooled 54 potential items based on theoretical considerations, content validity and psychometric properties [e.g., (66)]. Selected items related to loneliness and isolation (e.g., state/trait: “I lack companionship.”), social support and connectedness (state: “There are people I can talk to.”), social withdrawal (trait: “I like spending a lot of time by myself.”), extraversion (trait: “I am an outgoing person.”), and specific characteristics of social contacts in the context of social distancing, like the contact to people from the high-risk group or the use of virtual communication (state: “I maintain contacts via telephone/internet/app.”). Items for the assessment of state and trait loneliness and isolation were taken from or inspired by the (revised) University of California Los Angeles (UCLA) Loneliness Scale (4, 8) and the Social Isolation Scale (31). For the assessment of social support and connectedness as well as social anxiety and avoidance, we consulted the Multidimensional Scale of Perceived Social Support [MSPSS; (67)], the Social Interaction Anxiety Scale [SIAS; (46, 68)], and the Social Avoidance and Distress Scale [SADS; (69)]. Some items were modified to improve their fit (e.g., inversed phrasing). Lastly, we constructed items relating to sociodemographic and behavioral indicators of isolation and the social distancing context (see also Supplementary Table 1). These items considered individual differences in the need for social contact (e.g., trait: “Regular contact is important to me.”) and the need for face-to-face interactions (e.g., trait: “It is good for me to talk to friends and family in person.”). We also included items to assess (a lack of) contact to people from high-risk groups (state: “I miss the personal contact with people belonging to the high-risk group.”).

The resulting scale consisted of a state and a trait section (i.e., subscale). The pooled items were assigned to the state or trait subscale based on theoretical considerations, being considered more appropriate to measure acute (state) aspects and effects of the social distancing context (e.g., “I am unhappy being so withdrawn.”) or dispositional (trait) aspects like extraversion (e.g., “I am an outgoing person.”). In the state section, participants are asked via instruction to indicate how each item describes their feelings and experiences “at the current time” (e.g., “I'm alone too often.”, “There are people I can talk to.”). In the trait section, participants are asked to indicate how each item describes their feelings and experiences “in general,” not (only) at the current time (e.g., “I am lonely.”, “No one really knows me well.”). The items are evaluated on a 5-point Likert-scale ranging from “strongly agree” to “strongly disagree”. Items were included, modified or excluded based on multiple evaluations. The authors and a panel of five psychologists and five laymen of different ages (Min = 26, Max = 72) evaluated the items regarding their redundancy, clarity and relevance in the context of social distancing (i.e., current COVID-19-related restrictions in March 2020). The evaluation panels were interviewed on their thoughts about each scale item, screened for possible misunderstandings, and asked for reasons for their responses. Based on their feedback, one item was deleted, four were modified, one was replaced by a similar item with a better fit, and four items were added (e.g., to assess an individual's disposition to attend social events). Three loneliness and isolation items from the state scale were also added to the trait scale in response to the panel's feedback. The final scale for validation by exploratory factor analysis (EFA) consisted of 40 items (17 state, 23 trait items; see Supplementary Table 1 for the complete item list and their sources). This scale was once again presented to the panel group to validate that the changes actually responded to their critical suggestions.

### Validation of the LISD Scale

#### Samples

For the validation of the LISD scale, we collected data from two independent samples and conducted an exploratory (EFA) and a confirmatory factor analysis (CFA). Data were collected online using German nationwide (www.clickworker.com) or local online platforms. The survey targeted the general population without specific requirements. After the first COVID-19-related, lockdown in Germany, the first sample completed the LISD scale within 2 weeks under mild social distancing restrictions (starting from June 25, 2020). The second sample completed the scale under stricter COVID-19-related, lockdown conditions, i.e., under tightened social distancing restrictions (starting from December 11, 2020). This allowed us to test the reliability of the scale in two independent samples across two different situational contexts. Exclusion criteria were age <18, text input without meaning, insufficient data as indicated by response bias (e.g., straight-lining), a statement by the participant (validation question), very small (speeding) or large answering times as indicated by the median-based relative speed index [TIME_RSI ≤ 2; see (70)], and additional attention check questions (71). Multivariate outliers (Sample 1: *n* = 15; Sample 2: *n* = 14) in the LISD scale were identified via Mahalanobis distance (Sample 1: χ^2^ [40] = 73.40; Sample 2: χ^2^ [30] = 59.70) and excluded [threshold = 0.001; (72)].

For the first sample, we collected data from 343 adults. Ninety-nine participants had to be excluded based on the criteria above, including four dropouts. For the second sample, we collected data from 361 adults and excluded 57 data sets, including 12 dropouts and three participant exclusions due to failed attention checks (see Table 1 for sample characteristics and Supplementary Table 7 for an extended sample comparison). Sample size considerations were based on recommendations from the literature, e.g., minimum sample sizes of 100 (73) to 200 (74) or a recommended five to 10 observations per estimated parameter (73, 75). The final sample sizes are considered sufficient for EFA and CFA analyses [see e.g., (72, 73)]. While no statistical a priori power analysis was conducted, sensitivity power analyses with α = 0.05 and power (1-β) = 0.80 showed that both samples were large enough to detect small single regression effects with effect sizes of *f*^2^ = 0.03 (*t* = 1.97).

**Table 1:** Characteristics of study samples.

	**Sample 1 (*N* = 244)**	**Sample 2 (*N* = 304)**	**Group comparison**	***p*-value**
Age (SD)	28.65 (10.59)	40.52 (12.06)	*t*(540.16) = −12.24	<0.001
Female^a^	79.1%	37.2%	χ^2^(3) = 96.86	<0.001
Employed	45.1%	69.1%	χ^2^(1) = 31.10	<0.001
Student	55.3%	18.8%	χ^2^(1) = 77.97	<0.001
Average number of contacts per day (SD)	13.58 (33.66)	6.83 (18.46)	*t*(347.57) = 2.78	0.006
Stayed at home to avoid social contacts [last 2 weeks]^b^ (SD)	3.12 (1.19)	4.01 (0.99)	*t*(472.57) = −9.37	<0.001
Avoided physical contact [last 2 weeks]^b^ (SD)	4.37 (0.97)	4.65 (0.71)	*t*(432.28) = −3.82	<0.001

a *1 = identifying as female, 2 = identifying as male; no other gender identification option was chosen. ^b^Items range from 1 = “strongly disagree” to 5 = “strongly agree”*.

#### Measures of Mental Health, Social Support and Sociability

For the assessment of mental health, we used well-established clinical measures of anxiety and depression. Individual differences in anxiety were assessed using the trait scale of the State-Trait Anxiety Inventory [(76), STAI; (77)] and a 6-item short form of the STAI state scale (78). Individual differences in depression were assessed using the 2-item Patient Health Questionnaire [PHQ-2; (79)] and the simplified Beck Depression Inventory [BDI-V; (80)]. We included the MSPSS (67) and the SIAS (46, 68) as indicators of convergent validity for factors measuring social support or social anxiety and avoidance, respectively. As indicators of sociability and social engagement, we used the extraversion subscale of the NEO-Five Factor Inventory [NEO-FFI; (81, 82)] and the sociability subscale of the 10-item shyness and sociability scales for adults [German: “Schüchternheits- und Geselligkeitsskalen für Erwachsene,” SGSE; (83)].

### Data Analysis

#### Exploratory and Confirmatory Factor Analyses

All analyses were conducted in R [version 4.0.3; (84)]. We computed means, standard deviations, and ranges for the items and subscales of the LISD scale. There were no missing values as the online survey did not allow incomplete responses on the LISD scale and clinical questionnaires. For each clinical questionnaire, a total and (if applicable) subscale score was calculated for each participant. From this, we derived means, standard deviations, ranges, and indicators of internal consistency (Cronbach's α, McDonald's ω).

EFA was computed for the state and trait scale separately, similar to previous validations of instruments divided into a state and trait subscale with different instructions (85, 86). The EFA were calculated using the R package “psych” (87). We used principal axis factor analysis for factor extraction. We chose principal axis factor analysis because it is recommended for studies with the primary goal to identify latent dimensions (factors) represented in a scale's items (73). Moreover, it does not include distributional assumptions [e.g., multivariate normality; (88)], accounts for specific and error variance (73), and is robust regarding unequal factor loadings or factors with few indicators (89, 90). The CFA on the resulting factor solutions (2 state factors and 3 trait factors) were also computed separately using the R package “lavaan” (91). For EFA, the determination of number of factors and dimensionality of the LISD scale was guided by parallel analysis and minimum average partial (MAP)-test, and supported by inspection of the scree plot (72, 92). Oblique rotation (promax) was applied to account for correlated factors. Initial assumption checks of EFA and CFA included the Bartlett test of sphericity (*p* <0.05), the Kaiser Meyer Olkin criterion (KMO, or Measure of Sampling Adequacy [MSA], > 0.50; (73, 93)), and tests for acceptable multivariate normal distribution and linearity of the data (72). The determinant of the item correlation matrix was assumed to be small, but > 0.00001. The proportion of very small (*r* > 0.30) and very large (*r* > 0.70) correlations in the bivariate item correlation matrix was checked to exclude singularity and multicollinearity, respectively (93, 94). Items with a skewness > 2.0 (*n* = 2) were excluded from factor analysis (95).

Several criteria determined a stepwise item reduction throughout EFA. First, items with communalities *h*^2^ ≤ 0.20 were excluded from the unrotated factor solution (96, 97). After rotation, items with primary factor loadings of <0.35 were also removed, as recommended for sample sizes of approximately *N* = 250 (73). In case of multiple factor loadings, items with a difference between loadings of Δ <0.20 were excluded when also showing a communality of *h*^2^ <0.50 (72, 73). We computed internal consistency (Cronbach's α) for each LISD factor. Cronbach's α coefficient estimates the total variation in the scale shared by the included items. Values above 0.70 are considered acceptable indicators of overall scale reliability (98, 99). Items were excluded throughout the EFA if their exclusion considerably increased Cronbach's α (93). Lastly, we considered item discrimination (exclusion criteria: *r*_it_ <0.30) and item difficulty (exclusion criteria: *P* <0.20 and *P* > 0.80) within each factor (100–102). There were a number of borderline exclusion indications, i.e., items with values just above or below an exclusion threshold (e.g., communality), especially on the trait scale which originally contained more items than the state scale (23 vs. 17 items, respectively). In these cases, the exclusion decision was based on (1) the unambiguousness of the other exclusion criteria and (2) the individual item's value with regard to the scale's content and to its factor. That is, if there was not just one but several marginal exclusion criteria for one item, it was more likely to be excluded. In addition, items were not excluded if this would result in too few items per factor [a minimum of three items is recommended; (73)].

The two (state and trait) factor matrices resulting from EFA were then considered for factor content interpretation and labeling (72, 73). The comparative fit index [CFI; (103)], root mean square error of approximation [RMSEA; (104)] and root mean square of the residuals [RMSR; (105)] served as model fit indices. For CFA, we inspected the standardized root mean square of the residuals [SRMR; (106)]. CFI values > 0.95 indicate reasonable model fit (106), but a more liberal cutoff of 0.90 is also frequently accepted (107, 108). For RMSEA, RMSR and SRMR, low values are desirable. RMSEA values <0.06 indicate excellent fit and values <0.10 moderate fit (109, 110). RMSR values ≤ 0.08 and SRMR values <0.10 are acceptable (106). CFA target models were the 2-factor solution (12 items) for the state scale and the 3-factor solution (14 items) for the trait scale from EFA. Maximum likelihood (ML) estimation was applied. For CFA, we also examined the 90% confidence interval of the RMSEA (98) and modification indices. A χ^2^ difference test was calculated for comparison of the CFA target models to a 1-factor-model (state and trait total score, respectively), and to an alternative trait model resulting from inspection of modification indices. The corresponding Akaike information criterion (AIC) and Bayesian information criterion (BIC) are reported. The use of modification indices is a data-driven approach recommended to respecify models with poor fit, but needs to be carefully applied and theoretically justified (108, 111, 112).

The factor correlations provided by EFA and CFA represent the relations between state and trait loneliness and social isolation, social support, social interaction anxiety and extraversion in the context of social distancing. Due to related item exclusions throughout EFA, the influence of virtual communication was considered minor and not further investigated. Additionally, for inspection of convergent and discriminant validity of the LISD scale, and the role of social support and connectedness, sociability, and social interaction anxiety, we calculated Pearson correlations of the LISD factors with the relevant questionnaire scores described above (MSPSS; extraversion subscale of NEO-FFI; sociability subscale of SGSE; SIAS). These relations also served as justifications for the factor labels chosen based on the EFA factor solution. As one factor resulting from factor analyses (i.e., state factor 2) included an item taken from the MSPSS (“There is a special person with whom I can share my joys and sorrows.”), we excluded this item from the MSPSS sum score for this single correlation to avoid an artificially high correlation.

#### Regression Analyses

To assess the predictive strength of the LISD scale for clinical outcome variables, we calculated multiple regressions, using the R-package “stats” (84). Results were visualized with the “ggplot2” package (113). Variance inflation factors (VIFs) were calculated with the “car” package (114) to check for collinearity (115). For each target variable (i.e., depression and anxiety), three regression models with decreasing parsimony were compared. Model fit was compared using Analysis of Variance (ANOVA). Predictor variables for model 1 were the five LISD factors without interactions; for model 2 the LISD factors, age, gender, and social distancing compliance without interactions; and for model 3 the LISD factors, age, gender, social distancing compliance, and their interactions. All continuous predictors in our regressions were z-standardized (age, questionnaire scores, LISD factors). The remaining two categorical predictors were converted into binary items: Gender (identifying as female/male; no other option selected) and compliance to social distancing (yes/no). We created the two outcome variables from standardized state questionnaire scores based on their construct's theoretical relation and their Pearson correlations in the present sample. STAI state anxiety serves as the outcome variable “anxiety”. The outcome variable “depression” represents the mean score of PHQ-2 and BDI-V (*r* = 0.72, *p* <0.001).

## Results

### Exploratory Factor Analysis

Two items from the state scale were excluded due to high skewness (> 2.0). All other assumptions were fulfilled satisfactorily (Bartlett: χ^2^[105] = 1484.39, *p* <0.001; MSA = 0.9).

For the state scale, the EFA led to a 2-factor-solution with 12 items (i.e., five items were excluded; for item means, standard deviations, and factor loadings, see Supplementary Table 2). Both parallel analysis and MAP test suggested two factors, supported by the visual inspection of the scree plot. The fit was satisfactory (CFI = 0.97, RMSR = 0.03, RMSEA = 0.06). The fit of the off-diagonal values was 0.99. The two factors correlated with *r* = 0.56. State factor 1 included nine items and explained 32% of variance. Based on its items which were partly inspired or taken from the UCLA Loneliness Scale (e.g., “I lack companionship.” “I feel isolated from others”), it was labeled “lonely and isolated”. State factor 2 included three items (e.g., “There are people I can talk to.”) and explained 18% of variance. We inverted this factor and labeled it “connected and supported”, representing that social relations have not deteriorated in the present context, but that there is someone to talk to and provide support (34, 67). Internal consistency was high for state factor 1 (α = 0.87, ω = 0.92) and acceptable for state factor 2 (α = 0.67, ω = 0.71).

For the trait scale, the EFA led to a 3-factor-solution with 14 items (i.e., nine items were excluded; for item means, standard deviations, and factor loadings, see Supplementary Table 3). Parallel analysis suggested three factors and the MAP test two factors, but the visual inspection of the scree plot also indicated a 3-factor solution. The fit was satisfactory (CFI = 0.97, RMSEA = 0.06, RMSR = 0.03). The fit of the off-diagonal values was 0.99. Trait factors 1 and 2 correlated with *r* = −0.34, factors 1 and 3 with *r* = 0.67, and factors 2 and 3 with *r* = −0.50. Trait factor 1 included five items and explained 24% of variance. Based on its items which were partly inspired or taken from the UCLA Loneliness Scale (e.g., “I am lonely”, “I feel left out”.), it was labeled “loneliness and isolation”. Trait factor 2 included five items (e.g., “I find it easy to relax with other people”.), explained 19% of variance and was labeled “sociability and sense of belonging”. While *sociability* represents extraversion (42), a *sense of belonging* refers to generally feeling in tune and having a lot in common with the people one is surrounded by Lee and Cagle (34). Trait factor 3 included four items (e.g., “There is no one I feel close to.”) and explained 12% of variance. It was inverted and then labeled “social closeness and support”. Its items capture perceived general access to social *support* (67) and emotional *closeness*, i.e., feeling close to and known by one's social relations as opposed to having superficial relations (32, 34). Internal consistency was good for trait factor 1 (α = 0.85, ω = 0.87) and trait factor 2 (α = 0.82, ω = 0.85), and acceptable for trait factor 3 (α = 0.77, ω = 0.80).

All items provide acceptable discrimination and difficulty indices (see Supplementary Tables 2, 3). Factor correlations of EFA and CFA are presented in Table 2. Correlations regarding construct and criterion-related validity of the LISD scale were considered sufficient to continue to CFA. Items on virtual communication were excluded throughout EFA (e.g., state item: “I keep in touch via telephone/internet/app.”; trait item: “It is good for me to talk to friends and family via telephone/internet/app.”; exclusion criteria: communality <0.20).

**Table 2:** Pearson correlations of the five LISD factors for the EFA (Study 1, *N* = 244) and CFA (Study 2, *N* = 304) sample.

**LISD factor**	**Study**	***M***	***SD***	**α**	**Ω**	**State 1**	**State 2**	**Trait 1**	**Trait 2**
State 1: lonely & isolated	1	2.47	0.84	0.88	0.92	-			
	2	2.81	0.87	0.90	0.93				
State 2: connected & supported	1	4.31	0.72	0.67	0.71	−0.51***	-		
	2	3.79	0.82	0.62	0.68	−0.54***			
Trait 1: loneliness & isolation	1	2.13	0.81	0.85	0.87	0.66***	−0.63***	-	
	2	2.61	0.93	0.87	0.88	0.73***	−0.60***		
Trait 2: sociability & sense of belonging	1	3.85	0.73	0.82	0.85	−0.08	0.29***	−0.37***	-
	2	3.28	0.78	0.83	0.87	0.23***	0.12*	−0.14*	
Trait 3: social closeness & support	1	4.25	0.70	0.77	0.80	−0.35***	0.63***	−0.67***	0.44***
	2	3.73	0.84	0.81	0.84	−0.25***	0.60***	−0.56***	0.49***

LISD = Loneliness and Isolation during Social Distancing Scale. *p <0.05, ***p <0.001.

### Confirmatory Factor Analysis

The state and trait factor models resulting from EFA were tested via CFA in an independent sample. The initial assumption check for CFA provided satisfactory results. For CFA of the 2-factor state model, the fit indices were CFI = 0.86, SRMR = 0.08 and RMSEA = 0.13, with a 90% confidence interval of 0.12 to 0.14. The comparison with the 1-factor-model showed a significant difference in χ^2^ (χ^2^_diff_ (1) = 14.62, *p* <0.001), with AIC_2−factor_ = 9775.9 compared to AIC_1−factor_ = 9788.6 and BIC_2−factor_ = 9913.5 compared to BIC_1−factor_ = 9922.4. The state items and their means, standard deviations, and factor loadings are presented in Supplementary Table 4. Cronbach's α and McDonald's ω are 0.90 and 0.93 for state factor 1, and 0.62 and 0.68 for state factor 2, respectively.

In the CFA of the 3-factor trait model, one item (“I lack companionship.”) from trait factor 1 produced several high modification indices. It was removed after careful consideration (e.g., closeness in content to other factor 1 items; lowest factor loading on factor 1; cross-loading and low communality in EFA; equivalent state item with higher properties). The resulting model showed a significant difference in χ^2^ (*p* <0.001) compared to the original model and was therefore selected (AIC_reduced_ = 9925.3, AIC_original_ = 10718.6; BIC_reduced_ = 10081, BIC_original_ = 10886). The fit indices were CFI = 0.92, SRMR = 0.09 and RMSEA = 0.09, with a 90% confidence interval of 0.08 to 0.11. The trait items and their means, standard deviations, and factor loadings are presented in Supplementary Table 5. The 1-factor solution provided poor fit indices (CFI = 0.53, SRMR = 0.19, RMSEA = 0.22) and further model comparison was therefore discarded. Cronbach's α and McDonald's ω are 0.87 and 0.88 for trait factor 1, 0.83 and 0.87 for trait factor 2, and 0.81 and 0.84 for trait factor 3.

Factor means, standard deviations and correlations for the first (EFA) and second (CFA) sample are shown in Table 2. Indicators of loneliness and isolation (state 1, trait 1) correlate positively with each other and negatively with both being *supported and connected* (state 2) as well as *social closeness and support* in general (trait 3; all *p* <0.001, see Table 2 for *r*-values). Correlations notably differ between samples only on trait factor 2 (*sociability and sense of belonging*). Here, the correlation with being *lonely and isolated* (state 1) is positive in Sample 2 (*r* = 0.23, *p* <0.001) but non-significant in Sample 1 (*r* = −0.08, *p* = 0.189). In contrast, Sample 2 shows weaker correlations of *sociability and sense of belonging* with being *connected and support* (state 2; Sample 1: *r* = −29, *p* <0.001, Sample 2: *r* = 0.12, *p* = 0.043) and trait *loneliness and isolation* (trait 1; Sample 1: *r* = −0.37, *p* <0.001, Sample 2: *r* = −0.14, *p* = 0.013).

Finally, we inspected selected correlations to validate the LISD factors' labeling and convergent validity (see also Supplementary Table 6 for a complete list of correlations for convergent and discriminant validity). Factors indicating social support, connectedness and closeness correlated positively with perceived social support (e.g., state factor 2 [*connected and supported*] and MSPSS, *r* = 0.57, *p* <0.001; item “There is a special person with whom I can share my joys and sorrows.” excluded from MSPSS sum score) and extraversion (e.g., trait factor 3 [*social closeness and support*] and extraversion [NEO-FFI], *r* = 0.53, *p* <0.001). The convergent validity of trait factor 2 (*sociability and sense of belonging*) is represented in its high positive correlation with the Big Five's extraversion dimension (*r* = 0.80, *p* <0.001) and the SGSE's sociability subscale (*r* = 0.76, *p* <0.001), as well as its negative correlation with social interaction anxiety (SIAS; *r* = −0.68, *p* <0.001).

### Relationship Between LISD Scores and Mental Health Dimensions

The regression analyses presented below focus on the second sample (CFA; *N* = 304) as it represents a more heterogeneous sample (nationwide recruitment, see also Table 1). Most importantly, during this time of enhanced restrictions, social distancing compliance was higher and the number of daily social contacts was lower.

Model statistics, regression weights, effect sizes, and VIFs for multiple regression analyses with anxiety as outcome variable are reported in Table 3. The predictors for model 1 (LISD factors) and 2 (LISD factors, age, gender, social distancing compliance) showed acceptable VIFs below 5 which indicates low collinearity (115). In contrast, model 3 (LISD factors, age, gender, social distancing compliance, and their interactions) shows VIFs > 10. Moreover, model comparison (ANOVA) showed that the inclusion of interactions (model 3) did not improve model fit [model 1: *F* (18, 280) = 1.31, *p* = 0.178; model 2: *F* (15, 280) = 1.29, *p* = 0.211]. Model 3 is therefore not reported (but see Supplementary Table 9 for reports on all three models).

**Table 3:** Multiple regression analyses for predicting state anxiety.

	**Model 1**		**Model 2**			
**Model statistics**						
Adjusted *R*^2^	0.33		0.33			
F	30.57***		19.73***			
*(df)*	(5, 298)		(8, 295)			
	**Standardized regression weights (β), effect sizes (** ** $\eta_{\text{p}}^{2}$ ** **) and variance inflation factors (VIF)**					
	**β**	** $\eta_{\text{p}}^{2}$ **	**VIF**	**β**	** $\eta_{\text{p}}^{2}$ **	**VIF**
LISD state 1	0.26**	0.03	3.08	0.27**	0.03	3.26
LISD state 2	−0.10	0.01	2.09	−0.10	0.01	2.11
LISD trait 1	0.28**	0.03	3.32	0.26**	0.03	3.38
LISD trait 2	−0.25***	0.06	1.67	−0.25***	0.05	1.77
LISD trait 3	0.06	0.00	2.43	0.04	0.00	2.51
Age				0.01	0.00	1.10
Gender (female)				0.15	0.01	1.05
Compliance (yes)				−0.22	0.01	1.03

LISD state 1 = “lonely and isolated”; LISD state 2 = “connected and supported”; LISD trait 1 = “loneliness and isolation”; LISD trait 2 = “sociability and sense of belonging”; LISD trait 3 = “social closeness and support”; VIF = variance inflation factor. State anxiety was measured with a 6-item short form of the State-Trait Anxiety Inventory's (STAI) state scale (78). ** <0.01, *** <0.001.

**Figure 1:**
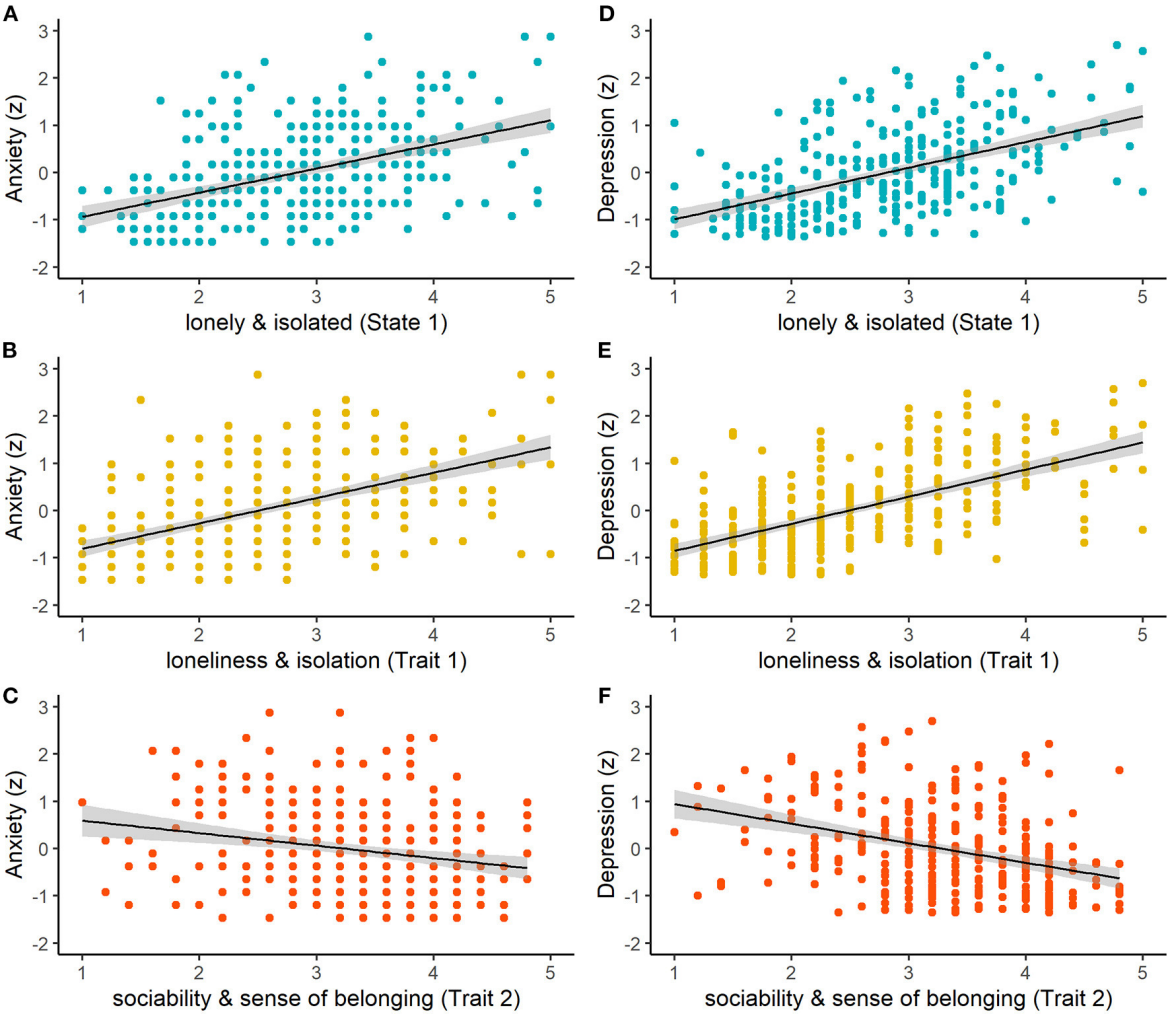
Relationships between raw LISD factor scores and z-standardized scores of anxiety (left column) and depression (right column) for **(A,D)** lonely and isolated (State 1; *p* = 0.002, *p* <0.001), **(B,E)** loneliness and isolation (Trait 1; *p* = 0.001, *p* = 0.020), and **(C,F)** sociability and sense of belonging (Trait 2; both *p* <0.001). The shaded areas indicate standard errors of the mean.

Regression analyses for model 1 revealed a significant positive relationship of anxiety with loneliness and isolation as a state (LISD state 1; β = 0.26, *SE* = 0.08, *p* = 0.002; Figure 1A) and trait (LISD trait 1; β = 0.28, *SE* = 0.09, *p* = 0.001; Figure 1B). Furthermore, anxiety was negatively related to trait *sociability and sense of belonging* (LISD trait 2; β = −0.25, *SE* = 0.06, *p* <0.001; Figure 1C). The LISD factors alone explained 32.8% of variance (adjusted *R*^2^) in state anxiety. The more complex model (model 2) did not improve prediction performance [*F* (3, 295) = 1.43, *p* = 0.234]. See Figure 1 for a visualization of significant predictors for anxiety.

Model statistics, regression weights, effect sizes, and VIFs for multiple regression analyses with depression as outcome variable are reported in Table 4. The predictors for model 1 and 2 showed acceptable VIFs below 5. Model 3 again showed some VIFs > 10 and did not improve model fit [model 1: *F* (18, 280) = 1.33, *p* = 0.165; model 2: *F* (15, 280) = 1.42, *p* = 0.135]. It is therefore not reported (but see Supplementary Table 11 for reports on all three models).

**Table 4:** Multiple regression analyses for predicting depression.

	**Model 1**		**Model 2**			
**Model statistics**						
Adjusted *R*^2^	0.51		0.51			
F	64.25***		40.43***			
*(df)*	(5, 298)		(8, 295)			
	**Standardized regression weights (β), effect sizes (** ** $\eta_{\text{p}}^{2}$ ** **) and variance inflation factors (VIF)**					
	**β**	** $\eta_{\text{p}}^{2}$ **	**VIF**	**β**	** $\eta_{\text{p}}^{2}$ **	**VIF**
LISD state 1	0.39***	0.10	3.08	0.38***	0.10	3.26
LISD state 2	−0.10^†^	0.01	2.09	−0.10^†^	0.01	2.11
LISD trait 1	0.16*	0.02	3.32	0.15*	0.02	3.38
LISD trait 2	−0.37***	0.16	1.67	−0.36***	0.15	1.77
LISD trait 3	−0.25	0.00	2.43	−0.04	0.00	2.51
Age				−0.01	0.00	1.10
Gender (female)				0.12	0.01	1.05
Compliance (yes)				−0.07	0.00	1.03

LISD state 1 = “lonely and isolated”; LISD state 2 = “connected and supported”; LISD trait 1 = “loneliness and isolation”; LISD trait 2 = “sociability and sense of belonging”; LISD trait 3 = “social closeness and support”; VIF = variance inflation factor. Depression is indicated by the mean score of the 2-item Patient Health Questionnaire [PHQ-2; (79)] and the simplified Beck Depression Inventory [BDI-V; (80)]. ^†^ <0.10, * <0.05, *** <0.001.

Regression analyses with the LISD factors as predictors (model 1) also revealed a significant positive relationship of depression with state 1 (β = 0.39, *SE* = 0.07, *p* <0.001; Figure 1D) and trait 1 (β = 0.16, *SE* = 0.07, *p* = 0.020; Figure 1E). A negative correlation was found with *sociability and sense of belonging* (LISD trait 2; β = −0.37, *SE* = 0.05, *p* <0.001; Figure 1F). Moreover, there was a marginally significant relationship with being *connected and supported* (LISD state 2; β = −0.10, *SE* = 0.05, *p* = 0.078). Once again, the more complex model (model 2) did not improve prediction performance [*F* (3, 295) = 0.87, *p* = 0.457]. The LISD factors explain 51.1% of variance in depression. See Figure 1 for a visualization of (marginally) significant predictors for depression.

Exploratory regression analyses with Sample 1 (EFA) underline the predictive strength of the LISD factors for mental health indices even under less severe social distancing conditions. The LISD factors explain 21.5% of variance in anxiety and 39.5% of variance in depression (see Supplementary Tables 8 and 10 for model statistics, regression weights, effect sizes, and VIFs).

## Discussion

This article presents the Loneliness and Isolation during Social Distancing (LISD) Scale, a measure for the assessment of loneliness and isolation during times of social distancing on the state and trait level. The final scale consists of 12 state and 13 trait items on five factors: *lonely and isolated* (state), *connected and supported* (state), trait *loneliness and isolation*, trait *sociability and sense of belonging*, and trait *social closeness and support*. Based on exploratory and confirmatory factor analyses, the factor solutions showed satisfactory fit in two independent and diverse samples. With the exception of *connected and supported*, all scales exhibit high reliability. In addition to convergent and discriminant validity assessed with established measures, state and trait LISD factors show strong predictive value for indicators of mental health, particularly depression. Our results underline our scale's adequacy for measuring mental strain in relation to loneliness and isolation. To the best of our knowledge, our LISD scale is the first to distinguish between state and trait aspects of loneliness and social isolation. Importantly, our analyses underline the expected gain in knowledge provided by the state-trait distinction. Extending previous findings lacking such a distinction, they imply a stronger mental health-depriving effect of state than trait loneliness and isolation in the context of social distancing.

### Scale Construction and Validity

The satisfactory fit indices derived from factor analyses in two heterogeneous samples are promising regarding the applicability of the LISD scale. The EFA's 2-factor state and 3-factor trait solutions' fit were confirmed in the second sample. The labeling of the state factor *lonely and isolated* and trait factor *loneliness and isolation* is supported by their items' origin from previous scales assessing loneliness and social isolation (e.g., the UCLA Loneliness Scale; 8). Some items even explicitly address loneliness and isolation, i.e., “I feel isolated from others.” and “I feel lonely.” The high positive correlation of perceived social support with state factor *connected and supported* and trait factor *social closeness and support* justifies their “social support” labeling. The distinction between “connected” and “closeness” is based on the remaining items' content (e.g., “I feel that my relationships with friends have deteriorated.” vs. “There is no one I feel close to.”) and their theoretical relevance for individual differences in loneliness and isolation (29, 31, 34). Note that despite high convergent validity, insufficient reliability of *connected and supported* implies that this factor may need additional items with higher reliability. The remaining factor *sociability and sense of belonging*'s items represent a tendency to seek social engagement and a feeling of belonging there. Convergent validity and support for its labeling as a “sociability” factor are provided in *sociability and sense of belonging*'s positive correlations with extraversion (NEO-FFI) and sociability (SGSE), and a negative correlation with shyness [i.e., a construct negatively associated with sociability; (116)]. Moreover, two of its items were previously used to measure social avoidance and distress [“I often find social occasions upsetting.”; “I find it easy to relax with other people.”, inverted coding; (69)]. Low scores on these items should therefore indicate a tendency toward social engagement.

The original scale for EFA contained a number of items that were included to target additional aspects related to social distancing. While other social distancing-specific items (e.g., “Regular contact is important to me.”) were excluded throughout EFA, the effect of the (lack of) contact to people from the high-risk group was included in the factor *lonely and isolated*. Despite their potential importance (53, 54, 117), items related to virtual communication (e.g., “I keep in touch via telephone/internet/app.”) were excluded due to low communalities. Future studies should investigate the potential effect of virtual communication further, using more objective measures of virtual interactions and contacts such as app usage times.

In accordance with previous literature (28), positive factor correlations imply that pre-existing general loneliness and social isolation are a risk factor for feeling lonely and isolated in an acute context of social distancing. Furthermore, previous findings suggest that acute loneliness is dependent on the social context (118). Negative correlations between the LISD factors measuring social factors (e.g., *social closeness and support*) with the LISD factors measuring loneliness and isolation indicate that social support, connectedness and closeness protect against loneliness and isolation, both on a state and trait level. This is in accordance with research on social distancing measures throughout the COVID-19 pandemic (50). The researchers found a loneliness-increasing effect of having less than five close relations and a loneliness-decreasing effect of face-to-face interactions and longer and more frequent interactions with emotionally close relations (50). Notably, the LISD scale allows to assess these factors and relations in an economic way, using just 25 items while also distinguishing between dispositional and acute influences on the degree of acute loneliness and isolation (as well as anxiety and depression). In contrast, previous researchers had to use multiple measures [e.g., single items on sociodemographic and social network information combined with other questionnaires without state-trait distinction; (50)].

Although our findings imply a protective role of *sociability and sense of belonging* against trait loneliness and isolation as well as anxiety and depression, its relationship with state loneliness and isolation is less clear. Correlations between the LISD factors point in the same direction across both samples, with one exception: While playing a protective role in the exploratory first sample, *sociability and sense of belonging* were associated with higher *lonely and isolated* scores in the confirmatory second sample. The second sample's greater variance in sociodemographic factors, particularly their higher age, could contribute to this difference to the younger and more homogeneous first sample [see e.g., (119)]. However, note that the first sample was collected during milder social distancing, with less compliance to social distancing measures and a higher frequency of daily contacts. It is likely that more sociable individuals were still able to fulfill their need of social contacts to a sufficient level, thus feeling less lonely (29, 31). This could have been denied to the second sample due to strict restrictions which encouraged staying at home, closed public places, and discouraged group gatherings and contacts beyond households (120). As a result, more sociable individuals grew lonelier and more isolated as they could not satisfy their pronounced need for social engagement. Besides sociability, losing a sense of belonging due to restricted contacts could also play a role here. Although usually functioning as a protective factor against loneliness (40), it may be too strongly impaired by social distancing and therefore unable to protect against acute loneliness and isolation. Social interaction anxiety (SIAS) was associated with higher state and trait loneliness and isolation. However, the *sociability and sense of belonging* factor includes negatively loaded items representing social interaction anxiety, avoidance and distress. For instance, a high score for the factor's item “I often find social occasions upsetting.” led to lower *sociability and sense of belonging*, which in turn related to lower *lonely and isolated* scores in the second sample. A trait tendency to avoid social gatherings and to feel uncomfortable among other people could therefore protect against loneliness during strict contact restrictions (but not at times of milder restrictions). In line with previous findings regarding a nationwide lockdown (49), our findings imply that strict social distancing circumstances may have a stronger impact on more sociable and socially integrated persons regarding acute loneliness and isolation. Thus, social distancing may overshadow the generally loneliness-reducing effects of sociability, extraversion, and a sense of belonging (44, 45).

### Prediction of Anxiety and Depression

The LISD scale shows strong predictive strength for mental health dimensions, i.e., anxiety and depression. The high proportions of explained variance (33% for anxiety, 51% for depression) revealed by multiple regression analyses show that in the context of social distancing, the 25-item LISD scale can predict mental health in an efficient way, particularly regarding increases in depression. Regression models with just the five LISD factors as predictors showed that in a phase of strict social distancing measures (Sample 2), being *lonely and isolated* predicts higher state anxiety and depression with a small and moderate effect, respectively. Higher *loneliness and isolation* on a trait level shows a similar, but smaller effect. Note that a measure without the state-trait distinction may have overlooked the strong effect of acute feelings of loneliness and isolation. In addition, the trait factor *sociability and sense of belonging* predicted lower levels of anxiety and depression, with a moderate and large effect, respectively.

Regression analyses show the value in distinguishing between state and trait aspects of loneliness and isolation in the context of mental health. Both anxiety and depression increase with higher scores in *lonely and isolated*, and *loneliness and isolation*. This risk-enhancing role of loneliness and isolation for anxiety and depression is in accordance with previous literature (3, 6), including research involving social distancing (15, 19). In addition, however, our results imply a higher predictive strength of state compared to trait loneliness and isolation for increases in depression during times of social distancing. This suggests a more important role of acute compared to perpetual loneliness and isolation in predicting mental health. Differentiating between state and trait aspects refines our understanding of loneliness and social isolation (121), as in other assessments of emotion and personality with a state-trait distinction, e.g., measurements of anxiety and anger (122).

In contrast to their positive relation with loneliness and isolation, anxiety and depression decrease with higher *sociability and sense of belonging* trait scores. The protective role of *sociability and sense of belonging* against depression and anxiety agrees with previous findings regarding a mental health-enhancing role of extraversion (45) and sense of belonging (40, 41). Note that the negative relation to depression and anxiety is in contrast to *sociability and sense of belonging*'s concurrent positive relation to feeling *lonely and isolated*. Based on our results, an individual who generally seeks and appreciates social contacts and feels like having a lot in common with the people around them (i.e., someone scoring high on *sociability and sense of belonging*) feels more lonely and isolated during strict social distancing conditions (than someone scoring low on *sociability and sense of belonging*). However, this person is also expected to report lower levels of depression and anxiety. Thus, although being linked to acute loneliness and isolation which usually relates to higher depression and anxiety, extraversion appears to retain its established mental health-enhancing effect (40, 41, 45) in a strict social distancing context. This may be supported by the positive correlation of *sociability and sense of belonging* with *social closeness and support*, which in turn is a protective factor against both state and trait loneliness and isolation and increased depression and anxiety.

Depression was marginally reduced by higher *connected and supported* scores. Apart from this, *connected and supported* and *social closeness and support* remained non-significant predictors in our regression models. This is surprising as social support was strongly associated with lower depression and anxiety in previous research (38, 40, 123). Despite this finding, we believe that identifying a lack of social support, closeness and connectedness (e.g., with low scores on the LISD scale's *connected and supported* and *social closeness and support* factors) is still relevant to both loneliness and mental health in the context of social distancing. Previous psychiatric research has underlined the crucial role of sufficient access to social contacts, activities, support and integration in protecting against loneliness and poor mental health (124, 125). The negative association of LISD factors assessing loneliness and isolation (state and trait) with the LISD factors assessing social connectedness, closeness and support (state and trait) visible in factor correlations implies that interventions supporting socialization could be effective in reducing loneliness and social isolation. Based on the present findings and previous research on the link between loneliness and mental health (6, 125), this should in turn lead to a lower risk of decreases in mental health. The support of socialization is particularly challenging in times of social distancing. It could be achieved by the enabling of appropriately distanced in-person meetings or by organized, targeted use of virtual communication tools (126–128), e.g., phone or video calls by volunteers (129). This may require social distancing-related tailoring of technology-based interventions, telemedicine consultations and teletherapy, and may even require the provision of technological devices to those lacking financial resources (117, 128–130).

The additional integration of other factors (i.e., compliance, gender, age) did not improve model performance, further supporting the scale's predictive value and economy. Although generally being associated with both loneliness and mental health, the inclusion of age and gender did not improve model fit. Against expectations, the individual compliance to social distancing also did not play a role in predicting anxiety and depression. However, the percentage of compliance was high (82.2%). Possibly, even if a person did not comply with social distancing, their social contacts did, exposing them to restricted social contacts all the same. Moreover, higher proportions of explained variance in the second sample compared to explorative regression analyses in the first sample underline the risk-enhancing role of social contact restrictions for mental health problems in the context of loneliness and isolation (123, 131, 132). Note, however, that the first and second sample also differed on other aspects (e.g., occupation), prohibiting definite conclusions on the effect of more strict contact restrictions.

The positive associations of LISD state and trait loneliness and isolation with depression and anxiety further establish loneliness and social isolation as crucial covariates of decreases in mental health (1, 2, 6), particularly in the context of social distancing (15, 131). At the same time, *sociability and sense of belonging* is associated with lower depression and anxiety levels (but also with higher state loneliness and isolation during strict social distancing conditions). However, based on our analyses, we cannot draw directional conclusions for these relationships. That is, for example, *sociability and sense of belonging* might not protect against depression, but be deprived by depression. Still, our findings underline the strong associations between depression and anxiety with loneliness, isolation, sociability, and a sense of belonging. Consequently, the LISD scale's assessment of state and trait indicators of loneliness, social isolation and associated factors could provide a more refined identification of loneliness-related covariates of poor mental health in clinical and therapeutic settings to better integrate them into therapeutic interventions [e.g., Internet-Based Cognitive Behavior Therapy for Loneliness; (133, 134)]. In the context of social distancing, increases in loneliness seem inevitable, and previous work has already highlighted the relevance of measuring loneliness and isolation in the context of social distancing for the protection and enhancement of wellbeing and mental health (65, 131). The LISD scale can be applied to identify those individuals particularly vulnerable to mental health-depriving effects of social contact restrictions, both on a dispositional and situational level (i.e., high levels of loneliness and isolation, low levels of sociability and sense of belonging). In the therapeutic setting, this could enable the clinician to individualize interventions by explicitly targeting these aspects. Besides factors directly related to the prediction of anxiety and depression, intervention strategies targeting the improvement of (perceived) social support, closeness and connectedness could reduce state and trait levels of loneliness and isolation. If successful, this could indirectly reduce the risk of decreases in mental health.

### Limitations and Outlook

As all new instruments, our scale, and in particular one of its factors (*connected and supported*), should be validated in independent studies. The LISD scale was constructed based on state-of-the-art criteria for item selection and scale validation (EFA, CFA). The state factor solution's SRMR indicates acceptable fit, while RMSEA and CFI lie slightly outside the targeted ranges. However, these criteria alone are not sufficient to make a general judgment about the quality of a scale (107, 108), and other indicators of validity (e.g., convergent validity) were satisfying. Each factor's internal consistency was acceptable or high after EFA, supporting their adequacy for the CFA analyses. After CFA, only one factor's reliability (*connected and supported*) was below the recommended value of > 0.70 (α = 0.62, ω = 0.68). This may have been due to the small number of items (i.e., three) and the breadth of the construct (i.e., social support and connectedness) that this short factor aims to measure (135). Importantly, although only with marginal significance, the factor *connected and supported* tended to predict (lower) depression levels along with the other factors of the scale. Note that although the labeling of the factors *lonely and isolated* and *loneliness and isolation* is supported by their items' adaption from the established UCLA Loneliness Scale, future studies should assess their convergent validity by including an independent loneliness measure.

Fluctuations of infection waves and related governmental restrictions including social distancing and nationwide lockdowns prevented data collection under equal social distancing restrictions for EFA and CFA, respectively. Moreover, the distribution of gender and the mean age (but not the age span) differed between samples. This could have reduced the applicability of the EFA factor solution to the second data set. However, despite the differences between the sample regarding age and the situational context (milder vs. more severe phase of the COVID-19 pandemic in Germany), our results show a good fit of the LISD scale. Although originally validated in a less heterogeneous sample with less social distancing behavior, the factor solution was still largely confirmed in the second sample, and the relation between LISD factors and mental health dimensions (i.e., depression, anxiety) could be replicated. Note however that our study targeted healthy participants. A validation of the factor structure in a clinical sample would allow further insight into the applicability among the general public, psychiatric patients and beyond. In addition, a longitudinal assessment under mild and strict social distancing conditions would allow more concise conclusions on the effect of the extent of social distancing, e.g., regarding the relation of *sociability and sense of belonging* with acute loneliness and isolation. This would also allow for a validation of the differentiation of state and trait items and factors via test-retest reliability. While trait factors should remain stable, we would expect higher variability in the two state factors.

Lastly, we would like to point out a limitation that frequently occurs in the literature when reporting results from different questionnaires. The questionnaires we used for the assessment of anxiety and depression utilize different instructions, therefore assessing feelings and experiences in slightly diverse time windows, i.e., “in the current moment” (STAI state), “over the last 2 weeks” (PHQ-2), or as a “current attitude toward life” (BDI-V). A common time frame would be more precise.

In conclusion, we developed and validated the Loneliness and Isolation during Social Distancing (LISD) Scale which assesses state and trait factors of loneliness and isolation in times of social distancing. For the first time, acute and dispositional aspects of loneliness and isolation can be measured in parallel and with just one instrument. Moreover, the LISD scale can help predict mental health outcomes, i.e., depression and state anxiety.

## Data Availability Statement

The final LISD Scale (English and German version) and the datasets presented in this study are publicly available in the following online repository: https://osf.io/ezdgt.

## Ethics Statement

The studies involving human participants were reviewed and approved by the Ethics Committee of the Institute of Psychology of the Faculty of Human Sciences, Julius-Maximilians-University of Würzburg, Würzburg, Germany. The participants provided their written informed consent to participate in this study.

## Author Contributions

MG, JH, JD, and GH contributed to conception and design of the scale and study. MG and LM programmed the survey and collected the data. MG and MW performed data curation and statistical analyses with input from LM and supervised by GH. MG and MW wrote the manuscript with input from GH. LM, JH, and JD contributed to the manuscript revision. All authors read and approved the submitted version.

## Funding

This study was supported by the German Research Foundation (HE 4566/5-1) and by the Volkswagenstiftung (Az 99451). This publication was supported by the Open Access Publication Fund of the University of Würzburg.

## Conflict of Interest

The authors declare that the research was conducted in the absence of any commercial or financial relationships that could be construed as a potential conflict of interest.

## Publisher's Note

All claims expressed in this article are solely those of the authors and do not necessarily represent those of their affiliated organizations, or those of the publisher, the editors and the reviewers. Any product that may be evaluated in this article, or claim that may be made by its manufacturer, is not guaranteed or endorsed by the publisher.
