# Supplementary material for: Construction and Validation of a Scale to Measure Loneliness and Isolation During Social Distancing and Its Effect on Mental Health
Source: Front Psychiatry. 2022 Apr 5;13:798596. doi: 10.3389/fpsyt.2022.798596 (PMC9017747; doi:10.3389/fpsyt.2022.798596)
Supplement: Supplementary file 1 [file Data_Sheet_1.pdf]

Supplementary Material

1 Original LISD items for exploratory factor analysis

We report the original items and their English translations of the 40 item-LISD scale prior to exploratory factor analysis (EFA) in Supplementary Table 1. The table contains information on each item's source. Items were taken from the Multidimensional Scale of Perceived Social Support (MSPSS; 1), the Social Avoidance and Distress Scale (SADS; 2), the University of California Los Angeles (UCLA) Loneliness Scale (3), its 6-item revised short form (4), and the German version of the revised UCLA Loneliness Scale (5)

Items of the 40-item W-LISD constructed for exploratory factor analysis.

SS	excl	Items in German	Items in English	source
S	x	Ich verbringe regelmäßig Zeit in Anwesenheit von Freunden und/oder Familie.	I regularly spend time in the company of friends and/or family.	s.c.
S		Ich bin unzufrieden darüber, dass ich so zurückgezogen sein muss.	I am unhappy being so withdrawn.	ULS-6
S		Ich habe Menschen, mit denen ich sprechen kann.	There are people I can talk to.	UCLA-R (g)
S		Ich fühle mich allein.	I feel alone.	UCLA-R (g)
S		Ich vermisse den persönlichen Kontakt zu Menschen, die zur Risikogruppe gehören.	I miss the personal contact with people belonging to the high-risk group.	s.c.
S		Ich fühle mich ausgeschlossen.	I feel left out.	ULS-6
S		Ich bin unglücklich darüber, dass ich so viele Dinge allein tun muss.	I am unhappy doing so many things alone.	UCLA
S	x	Ich habe niemanden, an den ich mich wenden kann.	There is no one I can turn to.	UCLA-R (g)
S		Ich warte darauf, dass die Leute anrufen oder mir schreiben.	I find myself waiting for people to call or write.	UCLA
S		Es gibt eine besondere Person, mit der ich meine Freuden und Sorgen teilen kann.	There is a special person with whom I can share my joys and sorrows.	MSPSS
S	x	Ich habe das Gefühl, dass sich meine Beziehung zu Familienmitgliedern verschlechtert hat.	I feel that my relationships with friends have deteriorated.	s.c.
S		Ich habe das Gefühl, dass sich meine Beziehung zu Freunden verschlechtert hat.	I feel that my relationships with family members have deteriorated.	s.c.
S		Ich bin von den anderen isoliert.	I feel isolated from others.	ULS-6
S	x	Ich habe Kontakt mit jemandem, der oder die mir nahesteht.	I am in contact with someone who I am close to.	s.c.

SS	excl	Items in German	Items in English	source
S		Mir fehlt die Gesellschaft Anderer.	I lack companionship.	ULS-6
S	x	Ich pflege Kontakte per Telefon/Internet/App.	I maintain contacts via telephone / internet / app.	s.c.
S		Ich bin zu viel allein.	I'm alone too much.	UCLA-R (g)
T		Ich bin ein geselliger Mensch.	I am an outgoing person.	UCLA-R (g)
T		Ich bin einsam.	I am lonely.	s.c.
T	x	Ich habe engen Kontakt zu Personen, die in der Corona-Pandemie zur Risikogruppe gezählt werden.	I am in close contact with people who are considered to be in the risk group in the Corona pandemic.	s.c.
T		Ich fühle mich niemandem nah.	There is no one I feel close to.	UCLA-R (g)
T	x	Es tut mir gut, mit Freunden und Familie persönlich zu sprechen.	It is good for me to talk to friends and family in person.	s.c.
T	x	Es tut mir gut, mit Freunden und Familie per Telefon/Internet/App zu sprechen.	It is good for me to talk to friends and family via phone / internet / app.	s.c.
T	x	Die Leute um mich herum haben ganz andere Interessen und Ideen als ich.	The people around me have very different interests and ideas than I do.	UCLA-R (g)
T	x	Ich kann mit anderen zusammen sein, wenn ich das will.	I can be with others if I want to.	UCLA-R (g)
T		Ich habe viel gemeinsam mit den Menschen um mich herum.	I have a lot in common with the people around me.	UCLA-R (g)
T		Ich fühle mich wohl, wenn ich unter Menschen bin.	I feel comfortable when I'm around people.	s.c.
T	x	Regelmäßiger Kontakt zu anderen ist mir wichtig.	Regular contact to others is important to me.	s.c.
T		Ich erhalte von meinen Freunden und/oder meiner Familie emotionale Hilfe und Unterstützung.	I get emotional help and support from my friends and/or family.	s.c.
T		Meine Freundschaften sind oberflächlich.	My friendships are superficial.	UCLA-R (g)
T		Niemand kennt mich wirklich gut.	No one really knows me well.	UCLA-R (g)
T	x	Ich habe einen Freundeskreis.	I have a group of friends.	UCLA-R (g)
T		Ich wünsche mir mehr Kontakt zu Menschen, mit denen ich reden kann.	I desire more contact with people I can talk to.	s.c.
T	x	Ich verbringe gerne viel Zeit alleine.	I like spending a lot of time by myself.	s.c.
T		Ich finde soziale Anlässe oft beunruhigend.	I often find social occasions upsetting.	SADS
T	x	Ich habe nicht den konkreten Wunsch, Menschen zu meiden.	I do not have a specific desire to avoid people.	s.c.
T		Es fällt mir leicht, mich mit anderen Menschen zu entspannen.	I find it easy to relax with other people.	SADS

SS	excl	Items in German	Items in English	source
T	x	Mir fehlt die Gesellschaft Anderer.	I lack companionship.	ULS-6
T		Ich fühle mich ausgeschlossen.	I feel left out.	ULS-6
T		Ich bin von den anderen isoliert.	I feel isolated from others.	ULS-6

Note. SS = subscale, excl = items excluded throughout EFA. MSPSS = Multidimensional Scale of Perceived Social Support; S = state subscale; SADS = Social Avoidance and Distress Scale, s.c. = self-constructed; T = trait subscale; UCLA = UCLA Loneliness Scale; UCLA-R (g) = Revised UCLA Loneliness Scale (German version); ULS-6 = 6 Item-Revised-UCLA.

2 LISD scale item characteristics from exploratory factor analysis

In this section, we report item means, standard deviations, factor loadings and additional item characteristics of the 2-factor solution for the LISD state scale and the 3-factor-solution for the LISD trait scale from EFA (*N* = 244).

2.1 Data analysis

All analyses were computed in R (version 4.0.3; 6). EFA was calculated with the psych package (7) for R. We computed means, standard deviations, range, skewness and kurtosis for the 26 items of the LISD scale after item reduction.

2.2 Results

Descriptive statistics on item level for the LISD state scale from EFA are presented in Supplementary Table 2.

Supplementary Table 2

Items, descriptive statistics, and factor loadings for the final items of the state scale of the Loneliness and Isolation during Social Distancing (LISD) Scale in the Exploratory Factor Analysis (EFA) sample (Sample 1, N = 244).

			Factor Loading[1]							
	Items in German	Items in English	F1	F2	*M*	*SD*	skew	kurt	discr	diff
1	Ich bin unzufrieden darüber, dass ich so zurückgezogen sein muss.	I am unhappy being so withdrawn.	**.79**	-.16	3.15	1.24	-.07	-1.03	.62	.63
2	Ich habe Menschen, mit denen ich sprechen kann.	There are people I can talk to.	.13	**-.83**	4.51	.77	-1.64	2.46	.59	.90
3	Ich fühle mich allein.	I feel alone.	**.54**	.42	2.20	1.10	.65	-.29	.76	.44
4	Ich vermisse den persönlichen Kontakt zu Menschen, die zur Infektions-Risikogruppe gehören.	I miss personal contact with people belonging to the high-risk group.	**.60**	-.23	3.07	1.33	-.14	-1.13	.40	.61

			Factor Loading[1]							
	Items in German	Items in English	F1	F2	*M*	*SD*	skew	kurt	discr	diff
5	Ich fühle mich ausgeschlossen.	I feel left out.	**.47**	.38	1.83	1.05	1.21	.77	.67	.37
6	Ich bin unglücklich darüber, dass ich so viele Dinge allein tun muss.	I am unhappy doing so many things alone.	**.80**	.05	2.43	1.32	.47	-1.02	.77	.49
7	Ich warte darauf, dass die Leute anrufen oder mir schreiben.	I find myself waiting for people to call or write.	**.37**	.17	2.15	1.09	.59	-.66	.44	.43
8	Es gibt eine besondere Person, mit der ich meine Freuden und Sorgen teilen kann.	There is a special person with whom I can share my joys and sorrows. [2]	.27	**-.71**	4.41	.97	-1.51	1.70	.42	.80
9	Ich habe das Gefühl, dass sich meine Beziehung zu Freunden verschlechtert hat.	I feel that my relationships with friends have deteriorated.	.29	**.45**	1.98	1.13	.94	-.14	.42	.80
10	Ich bin von den anderen isoliert.	I feel isolated from others.	**.46**	.28	1.93	1.02	.84	-.18	.60	.39
11	Mir fehlt die Gesellschaft Anderer.	I lack companionship.	**.84**	-.14	3.30	1.21	-.33	-.75	.69	.66
12	Ich bin zu viel allein.	I'm alone too often. [2]	**.52**	.35	2.15	1.15	.71	-.47	.70	.43

Note. [1] standardized. Bold numbers indicate which factor the item belongs to based on EFA. English items were taken from their original scales or [2] translated from their original German items.
F1 = state factor 1, F2 = state factor 2, skew = skewness, kurt = kurtosis, discr = item discrimination, diff = item difficulty.

Descriptive statistics on item level for the LISD trait scale from EFA are presented in Supplementary Table 3.

Supplementary Table 3

Items, descriptive statistics, and factor loadings for the final items of the trait scale of the Loneliness and Isolation during Social Distancing (LISD) Scale in the exploratory factor analysis (EFA) sample (Sample 1, N = 244).

			Factor Loading[1]								
	Items in German	Items in English	F1	F2	F3	*M*	*SD*	skew	kurt	discr	diff
13	Ich bin ein geselliger Mensch.	I am an outgoing person.	.19	**.81**	-.00	3.97	.93	-.47	-.50	.64	.79
14	Ich bin einsam.	I am lonely. [2]	**.74**	-.07	-.01	1.93	.94	.91	.66	.67	.39
15	Ich fühle mich niemandem nah.	There is no one I feel close to. [2]	.43	.11	**.51**	1.62	.89	1.41	1.23	.63	.32

			Factor Loading[1]									
	Items in German	Items in English	F1	F2	F3	*M*	*SD*	skew	kurt	discr	diff
16	Ich habe viel gemeinsam mit den Menschen um mich herum.	I have a lot in common with the people around me. [2]	-.04	**.47**	-.30	3.80	.83	-.32	-.43	.55	.77
17	Ich fühle mich wohl, wenn ich unter Menschen bin.	I feel comfortable when I'm around people. [2]	.16	**.94**	.03	3.96	.92	-.46	-.62	.74	.79
18	Ich erhalte von meinen Freunden und/oder meiner Familie emotionale Hilfe und Unterstützung.	I get emotional help and support from my friends and/or family. [2]	.17	-.05	**-.87**	4.34	.84	-1.16	.82	.53	.33
19	Meine Freundschaften sind oberflächlich.	My friendships are superficial. [2]	.25	-.11	**.30**	1.85	.90	.94	.65	.48	.37
20	Niemand kennt mich wirklich gut.	No one really knows me well.	.20	-.03	**.53**	1.89	1.02	.96	.13	.65	.38
21	Ich wünsche mir mehr Kontakt zu Menschen, mit denen ich reden kann.	I desire more contact with people I can talk to. [2]	**.63**	.11	.17	2.65	1.19	.30	-.80	.63	.53
22	Ich finde soziale Anlässe oft beunruhigend.	I often find social occasions upsetting.	.32	**-.45**	-.04	2.28	1.21	.62	-.59	.48	.74
23	Es fällt mir leicht, mich mit anderen Menschen zu entspannen.	I find it easy to relax with other people.	-.10	**.73**	.09	3.80	.93	-.41	-.12	.65	.75
24	Mir fehlt die Gesellschaft Anderer.	I lack companionship.	**.70**	.28	-.07	2.74	1.28	.19	-1.01	.52	.55
25	Ich fühle mich ausgeschlossen.	I feel left out.	**.85**	-.12	-.13	1.75	.92	1.16	.94	.71	.35
26	Ich bin von den anderen isoliert.	I feel isolated from others.	**.83**	-.12	-.07	1.57	.87	1.59	2.07	.70	.31

Note. [1] standardized. Bold numbers indicate which factor the item belongs to based on EFA. English items were taken from their original scales or [2] translated from their original German items.
F1 = trait factor 1, F2 = trait factor 2, F3 = trait factor 3, skew = skewness, kurt = kurtosis, discr = item discrimination, diff = item difficulty.

3 LISD scale item characteristics from confirmatory factor analysis

In this section, we report item means, standard deviations, and factor loadings of the 2-factor solution for the LISD state scale and the 3-factor-solution for the LISD trait scale from confirmatory factor analysis (*N* = 304).

3.1 Data Analysis

CFA was calculated with the package "lavaan" (8) for R. We additionally computed means and standard deviations for the final 25 items of the LISD scale.

3.2 Results

Descriptive statistics on item level for the two factors of the LISD state scale from CFA are presented in Supplementary Table 4.

Supplementary Table 4

Items, descriptive statistics, and factor loadings for the final items of the state scale of the Loneliness and Isolation during Social Distancing (LISD) Scale in the Confirmatory Factor Analysis (CFA) sample (Sample 2, N = 304).

			Factor Loading						
Items in German		Items in English	std.	non-std.	*SE* Factor	*M*	*SD*	skew	kurt
**State Factor 1: lonely & isolated**									
12	Ich bin zu viel allein.	I'm alone too often.[1]	.84	1.08	.06	2.74	1.28	.16	-1.02
3	Ich fühle mich allein.	I feel alone.	.87	1.04	.06	2.59	1.20	.36	-.68
6	Ich bin unglücklich darüber, dass ich so viele Dinge allein tun muss.	I am unhappy doing so many things alone.	.77	.95	.06	2.70	1.23	.13	-1.00
5	Ich fühle mich ausgeschlossen.	I feel left out.	.80	.93	.06	2.30	1.66	.51	-.64
10	Ich bin von den anderen isoliert.	I am isolated from others.	.75	.87	.06	2.81	1.16	-.07	-.84
7	Ich warte darauf, dass die Leute anrufen oder mir schreiben.	I find myself waiting for people to call or write.	.66	.74	.06	2.55	1.13	.33	-.62
11	Mir fehlt die Gesellschaft Anderer.	I lack companionship.	.69	.74	.06	3.33	1.07	-.23	-.70
1	Ich bin unzufrieden darüber, dass ich so zurückgezogen sein muss.	I am unhappy being so withdrawn.	.59	.66	.06	3.19	1.13	-.14	-.72

	Items in German	Items in English	Factor Loading std.	non-std.	*SE* Factor	*M*	*SD*	skew	kurt
4	Ich vermisse den persönlichen Kontakt zu Menschen, die zur Infektions-Risikogruppe gehören.	I miss personal contact with people belonging to the high-risk group for infection. [1]	.34	.38	.07	3.07	1.12	.11	-.77
**State Factor 2: connected & supported**									
9	Ich habe das Gefühl, dass sich meine Beziehung zu Freunden verschlechtert hat.	I feel that my relationships with my friends have deteriorated. [1]	-.61	-.70	.08	4.00	.96	-.76	.06
2	Ich habe Menschen, mit denen ich sprechen kann.	There are people I can talk to.	.66	.63	.11	3.87	1.19	-.82	-.35
8	Es gibt eine besondere Person, mit der ich meine Freuden und Sorgen teilen kann.	There is a special person with whom I can share my joys and sorrows.	.49	.58	.08	2.50	1.14	.46	-.54

Note. std. = standardized, skew = skewness, kurt = kurtosis. All items range from 1 to 5. This measure in nonproprietary and may be used without permission. English items were taken from their original scales or translated from their original German items.

Descriptive statistics on item level for the two factors of the LISD state scale from CFA are presented in Supplementary Table 5.

Supplementary Table 5

Items, descriptive statistics, and factor loadings for the final items of the trait scale of the Loneliness and Isolation during Social Distancing (LISD) Scale in the Confirmatory Factor Analysis (CFA) sample (Sample 2, N = 304).

	Items in German	Items in English	Factor Loading std.	non-std.	*SE* Factor	*M*	*SD*	skew	kurt
**Trait Factor 1: Loneliness & Isolation**									
25	Ich fühle mich ausgeschlossen.	I feel left out.	.89	1.04	.05	2.19	1.17	.71	-.43
26	Ich bin von den anderen isoliert.	I am isolated from others.	.87	.99	.05	2.28	1.15	.48	-.78
14	Ich bin einsam.	I am lonely. [1]	.81	.99	.06	2.35	1.21	.62	-.56
20	Ich wünsche mir mehr Kontakt zu Menschen, mit denen ich reden kann.	I desire more contact with people I can talk to. [1]	.63	.68	.06	2.31	1.12	.65	-.27

	Items in German	Items in English	Factor Loading std.	non-std.	*SE* Factor	*M*	*SD*	skew	kurt
**Trait Factor 2: Sociability & Sense of Belonging**									
13	Ich bin ein geselliger Mensch.	I am an outgoing person.	.85	.90	.05	3.33	1.06	-.23	-.54
17	Ich fühle mich wohl, wenn ich unter Menschen bin.	I feel comfortable when I'm around people. [1]	.83	.80	.05	3.32	.96	-.16	-.21
23	Es fällt mir leicht, mich mit anderen Menschen zu entspannen.	I find it easy to relax with other people.	.76	.73	.05	3.23	.96	-.25	-.33
22	Ich finde soziale Anlässe oft beunruhigend.	I often find social occasions upsetting.	-.57	-.66	.06	2.74	1.16	.23	-.66
16	Ich habe viel gemeinsam mit den Menschen um mich herum.	I have a lot in common with the people around me.[2]	.54	.50	.05	3.26	.94	-.32	-.00
	**Trait Factor 3: Social Support & Closeness**								
21	Niemand kennt mich wirklich gut.	No one really knows me well.	.85	.94	.06	3.11	1.08	-.11	-.65
15	Ich fühle mich niemandem nah.	There is no one I feel close to. [1]	.80	.90	.06	2.18	1.12	.68	-.39
19	Meine Freundschaften sind oberflächlich.	My friendships are superficial. [1]	.67	.67	.05	2.27	1.00	.44	-.37
18	Ich erhalte von meinen Freunden und/oder meiner Familie emotionale Hilfe und Unterstützung.	I get emotional help and support from my friends and/or family. [1]	-.56	-.57	.05	3.68	.97	-.42	-.17

Note. std. = standardized, skew = skewness, kurt = kurtosis. All items range from 1 to 5. This measure in nonproprietary and may be used without permission. English items were taken from their original scales or [1] translated from their original German items.

4 Convergent and discriminant validity

Supplementary Table 6 shows correlations between the LISD factors and questionnaire scores in the CFA sample to indicate convergent and discriminant validity of each factor. We report correlations with extraversion (subscale of the NEO-FFI; 9, 10), sociability (subscale of the SGSE; 11), social support (MSPSS) and social interaction anxiety (SIAS; 12, 13).

Supplementary Table 6

Pearson correlations of the five LISD factors with questionnaire scores.

Variable	*M*	*SD*	state 1	state 2	trait 1	trait 2	trait 3
Extraversion (NEO-FFI)	4.31	.72	.01	.22**	-.28***	.80***	.53***
Sociability (SGSE)	3.79	.82	.25***	.02	-.05	.76***	.42***
MSPSS	2.13	.81	-.23***	.57***[1]	-.49***	.42***	.77***
SIAS	3.73	.84	.12*	-.28***	.41***	-.69***	-.51***

Note. LISD = Loneliness and Isolation during Social Distancing Scale; M = Multidimensional Scale of Perceived Social Support; NEO-FFI = NEO-Five Factor Inventory; SGSE = shyness and sociability scales for adults; SIAS = Social Interaction Anxiety Scale; state 1 = “lonely and isolated”; state 2 = “connected and supported”; trait 1 = “loneliness and isolation”; trait 2 = “sociability and sense of belonging”; trait 3 = “closeness and support”.
[1] item “There is a special person with whom I can share my joys and sorrows.” excluded from MSPSS sum score.
* indicates $p < .05$, ** indicates $p < .01$, *** indicates $p < .001$.

5 Comparison of sample characteristics from Sample 1 (EFA) and Sample 2 (CFA)

5.1 Objective

As the two samples for EFA (Sample 1) and CFA (Sample 2) were independently collected at different stages of the COVID-19 pandemic and related contact restrictions, we present a comparison of central sample characteristics below. Governmental safety restrictions included social distancing in public places at both time points. However, public life and social contacts were regulated to a much higher degree during Sample 2.

5.2 Measures

The online survey contained the initial version of the LISD scale consisting of 40 items. Besides basic sociodemographic variables and clinical questionnaires reported in the main manuscript, we assessed affectedness by the coronavirus (infection self- and others, perceived risk of infection), frequency of social contacts (direct and virtual), and acceptance of and compliance with social distancing measures.

5.3 Data analysis

For comparison of sample characteristics and questionnaire scores in Sample 1 and 2, we computed Welch two sample t-tests on relevant interval variables. Levene’s tests were calculated to test for

homoscedasticity and t-tests were adjusted accordingly. For categorical variables, χ^2 tests were computed to compare distributions.

5.4 Results

The EFA and CFA sample differed significantly in sociodemographic and clinical variables. The first sample was younger and reported more social contacts and less compliance to a subset of safety behaviors associated with social distancing (see Table 1 in main manuscript). The sample distributions did not differ in terms of education ($\chi^2(6) = 5.27$, $p = .510$). Hobbies ($\chi^2(5) = 24.33$, $p < .001$) were carried out more frequently in Sample 1, but the frequency of physical activity was distributed equally, "several times a week" being the most frequent answer (Sample 1: 48.3%; Sample 2: 38.7%). Concerning health in general, there was a sample difference ($\chi^2(4) = 31.25$, $p < .001$), with "very good" being indicated more frequently in Sample 1 (vs. "good" in Sample 2). Notably, 87.5% of Sample 1 and 79.9% of Sample 2 reported good or very good health, supporting the assumption that our online study advertisements mainly reached healthy samples. In accordance with the time course of the COVID-19 pandemic, known cases of infection with COVID-19 in private and/or work-related acquaintances were reported more frequently in Sample 2 (Sample 1: 31.2%; Sample 2: 44.1%; χ^2 (1) = 9.04, $p = .003$). A high majority of participants reported no previous or current self-infections (Sample 1: 96.7%; Sample 2: 97.7%; $\chi^2(2) = 1.72$, $p = .424$). In addition, participants perceived themselves more frequently as being at high risk of infection with COVID-19 in Sample 2 (Sample 1: Yes = 14.8%, No = 81.1%, Unsure/not specified = 4.1%; Sample 2: Yes = 19.7%, No = 68.8%, Unsure/not specified = 11.5%; χ^2 (2) = 13.78, $p = .001$).

Mental health was higher in the first compared to the second sample (Supplementary Table 5). The second sample showed higher state anxiety ($t(542.45) = -2.87$, $p = .004$). Differences in depression scores and trait anxiety were only marginally significant. Perceived social support was lower in Sample 2 ($t(544.32) = 6.43$, $p < .001$). The two samples differed on all five LISD factors: state factor 1 and trait factor 1 scores (i.e., *loneliness and isolation*) were higher in Sample 2, state factor 2 and trait factors 2 and 3 (i.e., *social support, connectedness and belonging*) were lower (all $p < .001$).

Supplementary Table 7

Factor scores of the Loneliness and Isolation during Social Distancing (LISD) scale and clinical questionnaire scores in the EFA (Sample 1, N = 244) and CFA (Sample 2, N = 304) sample.

	EFA		CFA				
Variable	*M*	*SD*	*M*	*SD*	*t*	*df*	*p*
LISD State 1: lonely/isolated	2.47*	0.84	2.81*	0.87	-4.66	546	<.001
LISD State 2: supported/connected	4.31*	0.72	3.79*	0.82	7.89	541.89	<.001
LISD Trait 1: loneliness/isolation	1.98*	0.81	2.48*	0.98	-6.63	545.59	<.001
LISD Trait 2: sociability/sense of belonging	3.85*	0.73	3.28*	0.78	8.68	546	<.001

	EFA		CFA				
Variable	*M*	*SD*	*M*	*SD*	*t*	*df*	*p*
LISD Trait 3: support/connectedness	4.25*	0.70	3.73*	0.84	7.78	545.37	<.001
PHQ-2	1.70	1.50	1.95	1.58	-1.94	546	.053
BDI-V	26.39	16.15	29.05	19.66	-1.74	545.69	.083
STAI-State	10.56*	3.21	11.4*	3.68	-2.87	542.45	.004
STAI-Trait	41.78	10.27	43.56	12.01	-1.87	543.95	.062
SIAS	22.94*	11.28	25.88*	15.3	-2.40	541.63	.017
MSPSS	65.33*	11.28	58.15*	14.88	6.43	544.32	<.001

Note. * indicates significant difference between samples. BDI-V = Beck's Depression Inventory (V); MSPSS = Multidimensional Scale of Perceived Social Support; PHQ-2 = Patient Health Questionnaire (2 items); SIAS = Social Interaction Anxiety Scale; STAI-State = State subscale of the State-Trait Anxiety Inventory.

6 Comparison of regression analyses in Sample 1 (EFA) and Sample 2 (CFA)

In the main manuscript, we report two multiple regression models for each target variable, i.e., depression and anxiety, for the CFA sample (Sample 2). Three models were tested, but the third and most complex did not improve model fit and was therefore not reported. Predictor variables for model 1 were the five LISD factors without interactions; for model 2 the LISD factors, gender, and social distancing compliance without interactions; and for model 3 the LISD factors, gender, social distancing compliance, and their interactions. Here, we report the model statistics for all three regression models for the EFA (Sample 1) and CFA (Sample 2) sample.

Supplementary Table 8 shows the model statistics, regression weights, and effect sizes for all three regression models predicting anxiety in the EFA sample. Supplementary Table 9 shows the model statistics, regression weights, and effect sizes for all three regression models predicting anxiety in the CFA sample. As for the CFA sample (see main manuscript), model comparison (ANOVA) in the EFA sample showed that the inclusion of gender, age, and social distancing compliance (model 2)= did not improve model fit, $F(3, 234) = .01$, $p = .998$. The inclusion of interactions (model 3) did not improve model fit compared to model 1 with just the LISD factors ($F(18, 219) = 1.45$, $p = .109$), but showed better fit than model 2 ($F(15, 219) = 1.64$, $p = .045$).

Supplementary Table 8

Multiple regression analyses for predicting anxiety the EFA sample (Sample 1, N = 244).

	Model 1	Model 2	Model 3
Model statistics			
Adjusted R^2	.216	.206	.242
F	14.3	8.83	4.36
(df)	(5, 237)	(8, 234)	(23, 219)

	Model 1			Model 2			Model 3		
Standardized regression weights (β), effect sizes (η_p^2), and variance inflation factors (VIF)									
	β	η_p^2	VIF	β	η_p^2	*VIF*	β	η_p^2	VIF
LISD state 1	.32***	.06	2.02	.32***	.06	2.16	.38	.08	18.52
LISD state 2	-.09	.01	2.05	-.09	.01	2.05	-.04	.01	18.20
LISD trait 1	.09	.00	3.13	.09	.00	3.22	.17	.00	19.80
LISD trait 2	-.11†	.01	1.30	-.11†	.01	1.34	-.10	.01	8.96
LISD trait 3	.00	.00	2.34	.00	.00	2.37	.12	.00	13.28
Age				-.01	.00	1.02	-.01	.00	1.10
Gender (female ♀)				-.01	.00	1.08	-.01	.00	1.41
Compliance (yes)				.02	.00	1.03	-.02	.00	1.14
Age x LISD state 1							.10	.01	2.19
Age x LISD state 2							.07	.00	2.51
Age x LISD trait 1							-.21†	.01	3.32
Age x LISD trait 2							-.20**	.04	1.22
Age x LISD trait 3							-.02	.00	2.35
LISD state 1 x ♀							.03	.00	15.45
LISD state 2 x ♀							.23	.00	15.48
LISD trait 1 x ♀							-.20	.00	16.14
LISD trait 2 x ♀							-.17	.00	7.80
LISD trait 3 x ♀							-.17	.00	13.11
LISD state 1 x C (yes)							-.10	.00	5.14
LISD state 2 x C (yes)							-.41*	.03	5.69
LISD trait 1 x C (yes)							.06	.00	7.89
LISD trait 2 x C (yes)							.20	.01	3.41
LISD trait 3 x C (yes)							-.01	.00	7.15

Note. LISD state 1 = "lonely and isolated"; LISD state 2 = "connected and supported"; LISD trait 1 = "loneliness and isolation"; LISD trait 2 = "sociability and sense of belonging"; LISD trait 3 = "closeness and support"; VIF = variance inflation factor.
† < .10, * < .05, ** < .01, *** < .001

Supplementary Table 9

Multiple regression analyses for predicting anxiety in the CFA sample (Sample 2, N = 304).

	Model 1	Model 2	Model 3
Model statistics			
Adjusted R^2	.328	.330	.340
F	30.57***	19.73***	7.80***
(df)	(5, 298)	(8, 295)	(23, 280)

	Model 1			Model 2			Model 3		
Standardized regression weights (β), effect sizes (η_p^2), and variance inflation factors (VIF)									
	β	η_p^2	VIF	β	η_p^2	VIF	β	η_p^2	VIF
LISD state 1	.26**	.03	3.08	.27**	.03	3.26	.84†	.03	29.10
LISD state 2	-.10	.01	2.09	-.10	.01	2.11	.21	.01	17.75
LISD trait 1	.28**	.03	3.32	.26**	.03	3.38	-.18	.03	28.72
LISD trait 2	-.25***	.06	1.67	-.25***	.05	1.77	-.47*	.06	21.51
LISD trait 3	.06	.00	2.43	.04	.00	2.51	.03	.00	38.87
Age				.01	.00	1.10	.01	.00	1.15
Gender (female ♀)				.15	.01	1.05	.18†	.01	1.09
Compliance (yes)				-.22	.01	1.03	-.36*	.01	1.27
Age x LISD state 1							.10	.01	3.03
Age x LISD state 2							.09	.01	2.27
Age x LISD trait 1							.04	.00	3.46
Age x LISD trait 2							.04	.00	1.83
Age x LISD trait 3							.03	.00	2.82
LISD state 1 x ♀							-.27	.01	5.36
LISD state 2 x ♀							-.05	.00	4.14
LISD trait 1 x ♀							.28	.01	5.64
LISD trait 2 x ♀							.07	.00	3.05
LISD trait 3 x ♀							-.04	.00	4.45
LISD state 1 x C (yes)							-.53*	.01	27.84
LISD state 2 x C (yes)							-.30	.01	18.83
LISD trait 1 x C (yes)							.40	.01	28.65
LISD trait 2 x C (yes)							.21	.00	19.91
LISD trait 3 x C (yes)							.04	.00	35.82

Note. LISD state 1 = "lonely and isolated"; LISD state 2 = "connected and supported"; LISD trait 1 = "loneliness and isolation"; LISD trait 2 = "sociability and sense of belonging"; LISD trait 3 = "closeness and support"; VIF = variance inflation factor.
† < .10, * < .05, ** < .01, *** < .001

Supplementary Table 10 shows the model statistics, regression weights, and effect sizes for all three regression models predicting depression in the EFA sample. Supplementary Table 11 shows the model statistics, regression weights, and effect sizes for all three regression models predicting depression in the CFA sample. As for the CFA sample (see main manuscript), model comparison (ANOVA) in the EFA sample showed that the inclusion of gender, age, and social distancing compliance (model 2) did not improve model fit, $F(3, 234) = .96$, $p = .601$. The inclusion of interactions (model 3) showed a tendency towards model fit improvement (model 1: $F(18, 219) = 1.54$, $p = .080$; model 2: $F(15, 219) = 1.71$, $p = .050$).

Supplementary Table 10

Multiple regression analyses for predicting depression in the EFA sample (Sample 1, N = 244).

	Model 1			Model 2			Model 3		
Model statistics									
Adjusted R^2	.395			.392			.419		
F	32.61			20.52			8.58		
(df)	(5, 237)			(8, 234)			(23, 219)		
Standardized regression weights (β), effect sizes (η_p^2), and variance inflation factors (VIF)									
	β	η_p^2	VIF	β	η_p^2	VIF	β	η_p^2	VIF
LISD state 1	.24***	.06	2.02	.23***	.05	2.16	.07	.05	18.52
LISD state 2	-.01	.00	2.05	-.01	.00	2.05	.12	.00	18.20
LISD trait 1	.19*	.02	3.13	.20*	.03	3.22	.41	.04	19.80
LISD trait 2	-.28***	.11	1.30	-.27***	.10	1.34	-.26*	.12	8.96
LISD trait 3	-.07	.00	2.34	-.08	.01	2.37	.02†	.01	13.28
Age				.03	.00	1.02	.04	.00	1.10
Gender (female ♀)				.14	.01	1.08	.19	.01	1.41
Compliance (yes)				.02	.00	1.03	.01	.00	1.14
Age x LISD state 1							.12*	.02	2.19
Age x LISD state 2							.19*	.03	2.51
Age x LISD trait 1							-.03	.00	3.32
Age x LISD trait 2							-.05	.00	1.22
Age x LISD trait 3							-.22**	.04	2.35
LISD state 1 x ♀							.15	.00	15.45
LISD state 2 x ♀							.08	.00	15.48
LISD trait 1 x ♀							-.09	.00	16.14
LISD trait 2 x ♀							-.15	.01	7.80
LISD trait 3 x ♀							-.16	.00	13.11
LISD state 1 x C (yes)							.05	.00	5.14
LISD state 2 x C (yes)							-.26†	.02	5.69
LISD trait 1 x C (yes)							-.18	.01	7.89
LISD trait 2 x C (yes)							.15	.01	3.41
LISD trait 3 x C (yes)							.01	.00	7.15

Note. LISD state 1 = “lonely and isolated”; LISD state 2 = “connected and supported”; LISD trait 1 = “loneliness and isolation”; LISD trait 2 = “sociability and sense of belonging”; LISD trait 3 = “closeness and support”; VIF = variance inflation factor.
† < .10, * < .05, ** < .01, *** < .001

Supplementary Table 11

Multiple regression analyses for predicting depression in the CFA sample (Sample 2, N = 304).

	Model 1			Model 2			Model 3		
Model statistics									
Adjusted R^2	.51			.51			.52		
F	64.25***			40.43***			15.29***		
(df)	(5, 298)			(8, 295)			(23, 280)		
Standardized regression weights (β), effect sizes (η_p^2), and variance inflation factors (VIF)									
	β	η_p^2	VIF	β	η_p^2	VIF	β	η_p^2	VIF
LISD state 1	.39***	.10	3.08	.38***	.10	3.26	.56	.10	29.10
LISD state 2	-.10†	.01	2.09	-.10†	.01	2.11	-.19	.01	17.75
LISD trait 1	.16*	.02	3.32	.15*	.02	3.38	-.04	.02	28.72
LISD trait 2	-.37***	.16	1.67	-.36***	.15	1.77	.43	.15	21.51
LISD trait 3	-.25	.00	2.43	-.04	.00	2.51	.28	.00	38.87
Age				-.01	.00	1.10	-.02	.00	1.15
Gender (female ♀)				.12	.01	1.05	.12	.01	1.09
Compliance (yes)				-.07	.00	1.03	-.15	.00	1.27
Age x LISD state 1							-.02	.00	3.03
Age x LISD state 2							-.05	.00	2.27
Age x LISD trait 1							-.01	.00	3.46
Age x LISD trait 2							.11	.02	1.83
Age x LISD trait 3							-.04	.00	2.82
LISD state 1 x ♀							-.05	.00	5.36
LISD state 2 x ♀							-.14	.01	4.14
LISD trait 1 x ♀							-.02	.00	5.64
LISD trait 2 x ♀							-.07	.00	3.05
LISD trait 3 x ♀							.09	.00	4.45
LISD state 1 x C (yes)							-.18	.00	27.84
LISD state 2 x C (yes)							.18	.00	18.83
LISD trait 1 x C (yes)							.24	.01	28.65
LISD trait 2 x C (yes)							.12	.00	19.91
LISD trait 3 x C (yes)							-.41	.01	35.82

Note. LISD state 1 = "lonely and isolated"; LISD state 2 = "connected and supported"; LISD trait 1 = "loneliness and isolation"; LISD trait 2 = "sociability and sense of belonging"; LISD trait 3 = "closeness and support"; VIF = variance inflation factor.
† < .10, * < .05, ** < .01, *** < .001

7 References

1. Zimet GD, Dahlem NW, Zimet SG, Farley GK. The Multidimensional Scale of Perceived Social Support. Journal of Personality Assessment. 1988;52(1):30-41.
2. Watson D, Friend R. Measurement of social-evaluative anxiety. J Consult Clin Psychol. 1969;33(4):448-57.
3. Russell D, Peplau LA, Cutrona CE. The revised UCLA Loneliness Scale: concurrent and discriminant validity evidence. J Pers Soc Psychol. 1980;39(3):472-80.
4. Neto F. Psychometric analysis of the short-form UCLA Loneliness Scale (ULS-6) in older adults. Eur J Ageing. 2014;11(4):313-9.
5. Döring N, Bortz J. Psychometrische Einsamkeitsforschung: Deutsche Neukonstruktion der UCLA Loneliness Scale. Diagnostica. 1993.
6. R Core Team. R: A language and environment for statistical computing. Vienna, Austria: R Foundation for Statistical Computing; 2020.
7. Revelle WR. psych: Procedures for personality and psychological research. 2017.
8. Rosseel Y. Lavaan: An R package for structural equation modeling and more. Version 0.5–12 (BETA). Journal of statistical software. 2012;48(2):1-36.
9. Borkenau P, Ostendorf F. NEO-FFI: NEO-Fünf-Faktoren-Inventar nach Costa und McCrae, Manual2008.
10. Costa PT, McCrae RR. Professional manual: revised NEO personality inventory (NEO-PI-R) and NEO five-factor inventory (NEO-FFI). Odessa, FL: Psychological Assessment Resources. 1992;61.
11. Asendorpf J. Schüchternheits-und Geselligkeitsskalen für Erwachsene. Berlin: Humboldt-Universität, Institut für Psychologie. 1997.
12. Heimberg RG, Mueller GP, Holt CS, Hope DA, Liebowitz MR. Assessment of anxiety in social interaction and being observed by others: The Social Interaction Anxiety Scale and the Social Phobia Scale. Behavior therapy. 1992;23(1):53-73.
13. Mattick RP, Clarke JC. Development and validation of measures of social phobia scrutiny fear and social interaction anxiety. Behav Res Ther. 1998;36(4):455-70.
